# Feasibility and acceptability of randomized controlled trial of intervention *vs* expectant management for early‐onset selective fetal growth restriction in monochorionic twin pregnancy

**DOI:** 10.1002/uog.29175

**Published:** 2025-01-24

**Authors:** A. Khalil, S. Prasad, J. J. Kirkham, R. Jackson, K. Woolfall, the FERN study group, A. Khalil, S. Prasad, J. J. Kirkham, R. Jackson, K. Woolfall, T. K. Mitchell, M. Popa, O. Yaghi, T. Ricketts, R. Ashcroft, C. Bailie, E. Blennerhasset, A. Breeze, A. Cameron, N. Fenwick, M. C. Haak, A. Healey, K. Hecher, S. Leven, E. Lopriore, S. Meher, J. Mendoza, T. Mousa, S. Newell, A. T. Papageorghiou, H. Samarage, J. Sandall, M. Turner, M. Watson

**Affiliations:** ^1^ Fetal Medicine Unit, Department of Obstetrics and Gynaecology St George's University Hospitals NHS Foundation Trust London UK; ^2^ Vascular Biology Research Centre, Molecular and Clinical Sciences Research Institute St George's University of London London UK; ^3^ Fetal Medicine Unit, Liverpool Women's Hospital University of Liverpool Liverpool UK; ^4^ Centre for Biostatistics, The University of Manchester Manchester Academic Health Science Centre Manchester UK; ^5^ Department of Statistics, Liverpool Clinical Trials Unit University of Liverpool Liverpool UK; ^6^ Institute of Population Health, Department of Public Health, Policy and Systems University of Liverpool Liverpool UK

## Introduction

Selective fetal growth restriction (sFGR) affects 10–15% of monochorionic twin pregnancies, leading to adverse perinatal outcomes such as stillbirth and cerebral palsy, with early‐onset cases posing significant management challenges^1^, ^2^. Should the smaller twin not survive, the cotwin faces an approximately 15% risk of death and a 26% risk of neurological disability^1^. For managing early‐onset sFGR, three primary strategies are considered: expectant management, which entails vigilant observation without direct intervention; selective termination of the smaller twin, to potentially improve the prognosis for the larger twin; and the use of placental laser photocoagulation, to disconnect the twins' shared blood vessels, aiming to mitigate risks associated with vascular connections. Each approach comes with its own set of risks and ethical dilemmas: expectant management can place a heavy emotional toll on parents due to the uncertainty of the twins' survival; selective termination might not align with some parents' values; and placental laser photocoagulation is technically challenging and has not yet been proved efficacious in sFGR cases as clearly as it has in other situations, such as twin‐to‐twin transfusion syndrome.

Management options, diagnostic criteria, monitoring protocols and gestational age at birth for cases of sFGR vary amongst fetal medicine units^3^, ^4^. To date, the management of these cases has been guided by results obtained from retrospective cohorts^5^, ^6^, ^7^, meta‐analyses^8^, ^9^ and guidelines^1^, ^10^, ^11^. In the Clinical Trials database, we found only one randomized controlled trial (RCT) that aimed to compare laser ablation and expectant management in monochorionic twins with sFGR^12^, ^13^, but the trial stopped recruitment prematurely. Our meta‐analysis in 2019^8^, encompassing 16 observational studies and 786 monochorionic twin pregnancies, examined perinatal outcomes according to management options and we concluded that, while expectant management was deemed reasonable for Type‐I sFGR, laser ablation in Type‐II/III sFGR was associated with higher mortality rates but lower morbidity. Therefore, we suggested a potential role for fetal therapy in severe cases of sFGR occurring at a gestational age far from viability. More recently, Buskmiller *et al*.^9^ compared perinatal outcomes following expectant management with those following laser ablation in a meta‐analysis which included six studies and 299 monochorionic pregnancies with sFGR. They reported that laser ablation was linked to a higher risk of fetal death for the growth‐restricted twin compared with expectant management (risk ratio (RR), 2.50; 95% CI, 1.43–4.37). However, laser treatment was associated with reduced abnormal neuroimaging findings in the normally growing twin (RR, 0.25; 95% CI, 0.07–0.97). Only one of the included studies was a RCT, while the others were observational cohorts, highlighting the need for an appropriately designed clinical trial to address this knowledge gap.

The National Institute for Health and Care Research (NIHR) Health Technology Assessment (HTA)‐funded FERN study, ‘Intervention or Expectant Management for Early Onset Selective Fetal Growth Restriction in Monochorionic Twin Pregnancy’, is a feasibility prospective mixed‐methods cohort study comprising three distinct work packages (WP), to determine the acceptability and feasibility of conducting a RCT comparing the outcomes of intervention *vs* expectant management for early‐onset sFGR in monochorionic twin pregnancies^14^. In this Opinion, we report the output of the FERN WP3 stakeholders' consensus meeting for determining the acceptability and feasibility of the proposed RCT. We also explore the feasibility of different study designs.

## Consensus meeting

### Recruitment

Key stakeholders, including both UK‐based and international FERN study grant co‐applicants, collaborators, principal investigators of the study sites, sponsor representatives, Patient and Public Involvement and Engagement (PPIE) representatives, trialists and study statisticians, as well as parents with experience of a monochorionic twin pregnancy with sFGR, were invited to participate in the consensus development meeting. The clinical stakeholders included both fetal medicine specialists and neonatologists.

### Organization and agenda of the meeting

**Figure 1:**
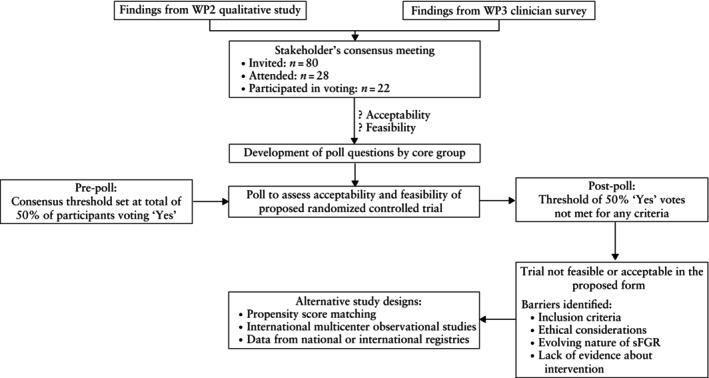
Flowchart summarizing proceedings of the FERN work package (WP) 3 stakeholders' consensus meeting for determining the acceptability and feasibility of a proposed randomized controlled trial comparing the outcomes of intervention *vs* expectant management for selective fetal growth restriction (sFGR) in monochorionic diamniotic twin pregnancies.

We invited stakeholders to a hybrid face‐to‐face/virtual platform meeting hosted at the Royal College of Obstetricians and Gynaecologists in London in July 2023. The meeting was led by the FERN project's Chief Investigator and facilitated by an independent member who was not involved in the project and abstained from voting. The meeting followed a prespecified agenda, which included brief presentations of the results from WP2 (qualitative study) and WP3 (clinician survey) (Figure 1). The core group developed questions regarding the acceptability (willingness of parents and clinicians to participate in a trial of this nature) and feasibility (e.g. ability to recruit and randomize participants to intervention, retention and generalizability) of a RCT comparing the outcomes of intervention *vs* expectant management for early‐onset sFGR in monochorionic twin pregnancies (Table S1). The questions were structured around several scenarios, including cases with abnormal or normal umbilical artery Doppler of the smaller twin and categorizations based on the type of sFGR (Type I, II or III). For each scenario, stakeholders were asked to vote on the acceptability and feasibility of conducting such a RCT, with options being ‘No’, ‘Unsure’ and ‘Yes’.

### Voting method

*A priori*, it was decided that if there were 50% or more votes of ‘Yes’ (for acceptability and/or feasibility) for each scenario, this would warrant further discussion about the plausibility of a trial. All stakeholders were treated as a unified group; i.e. the results were not categorized by stakeholder group. If the responses to all scenarios were not favorable (< 50% votes for acceptability and/or feasibility), then this would suggest that a trial was not acceptable or feasible. If particular scenarios were voted favorable (> 50% acceptability and/or feasibility) and others were not, then this was would suggest that a trial might be possible ‘under certain conditions’. The poll was conducted using Poll Everywhere software (https://www.polleverywhere.com/).

### Ethical approval and consent

The proceedings of this meeting were undertaken as part of the ongoing FERN study, which received ethical approval from the Health Research Authority Southwest – Cornwall and Plymouth Ethics Committee.

## Proceedings of the meeting (Figure 1)

Out of the 80 stakeholders invited to the meeting, 28 participated in the consensus meeting, of whom 22 participated in the voting (Table S2).

### Presentation of WP2: qualitative study results

This work package focused on qualitative research, including interviews and focus groups with parents and clinicians to explore trial design, acceptability, feasibility and decision‐making related to intervention or conservative management.

Participants agreed that it is essential to answer the research question for two purposes: (1) to enable clinicians to counsel parents confidently about management options for monochorionic twin pregnancy affected by sFGR; and (2) to provide parents with more information to help them in making difficult decisions with respect to an affected pregnancy. However, neither parents nor clinicians found a RCT of intervention *vs* expectant management to be acceptable in this case. A few clinicians expressed support for a RCT, but most agreed it would be challenging to recruit participants. Various factors that influence parents' and clinicians' decision‐making when the potential outcomes include death or serious disability include: inconsistency in clinical practice, the changing (evolving or inconsistent) nature of sFGR during pregnancy and the desire for more information before making decisions; the detailed findings from WP2 were published recently^15^.

### Presentation of WP3: clinician survey results

This international cross‐sectional survey of 113 clinicians aimed to identify current practices in managing sFGR in monochorionic twin pregnancies. For early‐onset sFGR in monochorionic diamniotic twin pregnancies, there was a general trend to manage Type‐I sFGR expectantly, with weekly surveillance. However, there was variation in the monitoring and management of Type‐II and Type‐III sFGR cases. Overall, the WP3 clinician survey demonstrated considerable variation in diagnosis, monitoring and management options and an unmet need to generate robust evidence by high‐quality research to address these uncertainties. The detailed findings from the WP3 clinician survey were published recently^16^.

### Poll to assess feasibility and acceptability of the proposed RCT

The poll results showed a clear consensus among stakeholders that it was neither acceptable nor feasible to conduct a RCT in the proposed format to evaluate the management of sFGR in these pregnancies (Table S1). The 50% ‘Yes’ threshold was not met for any scenario.

## Stakeholder views on barriers to delivering the definitive RCT and alternative study designs

Given the clear consensus among stakeholders that conducting a RCT in the proposed format to evaluate sFGR management was neither acceptable nor feasible, the discussion then focused on the barriers to delivering such a trial.

### Barriers to delivering the definitive RCT

Synthesis of the results from the three WPs in this meeting identified several barriers to delivering the trial. It may be possible to mitigate a few of these barriers, but, unfortunately, not all could be overcome. Some of the recurring themes/barriers that emerged during the discussion included:

1. Inclusion criteria: Type‐I sFGR should not be included in the proposed RCT as expectant management is nearly always the preferred option. There was marginal support for including Type‐II and Type‐III sFGR in the RCT.
2. Generalizability: the RCT could be designed with very specific inclusion/exclusion criteria, but this would be a highly selective RCT with reduced generalizability.
3. Lack of evidence about the efficacy and role of laser photocoagulation, especially the role of laser photocoagulation in the management of sFGR.
4. Evolving and sometimes inconsistent diagnosis of sFGR across gestation can make clinical decision‐making challenging.
5. Chance of crossover between trial arms.
6. Ethical considerations surrounding randomization and parental decision‐making, and the challenges of conducting a trial in this context.

These factors have the potential to impact negatively recruitment and randomization and subsequently affect patient retention in the proposed RCT, thereby significantly reducing trial feasibility. Since patient randomization and ‘testing’ of the protocol were not part of this feasibility study, these issues would require further exploration during a pilot phase incorporated into any subsequent trial.

### Alternative study designs

We discussed alternative study designs in which randomization is not necessary to answer the study question. However, the importance of RCTs as the gold standard for comparing treatments due to their comparison of two identical groups cannot be overemphasized.

The following categories of alternative study design were discussed:

1. Designs that include randomization but move the process away from randomization of individual patients, such as stepped‐wedge or expertise‐based designs^17^. These designs would still face the abovementioned challenges regarding selectivity and generalizability.
2. Designs that do not rely on randomization but aim to make causal statements, for example, propensity score analysis (an observational method that uses existing data, such as cohort or registry data, and does not involve randomization)^18^.
3. ‘Pseudo‐optimization’ or post‐intervention randomization^19^, a concept that involves randomizing patients after receiving a specific treatment or intervention. The challenge with pseudo‐randomization is that it may lead to high patient attrition rates if many patients do not fit the criteria for randomization.
4. Multicenter international prospective cohort study.

While considering the above study designs, other deliberations would include issues surrounding data quality control and data management, the choice of primary outcomes, both short‐term and long‐term, the use of composite outcome measures and data linkage with healthcare records as a potential strategy to address the rarity of the condition.

## Conclusions

The FERN stakeholders' group agreed that, while a RCT comparing the outcomes of intervention *vs* expectant management in early‐onset sFGR may not be feasible or acceptable in the proposed format, alternative study designs such as propensity‐score matching or an international multicenter observational cohort study may be plausible and appear promising to address this research question. The consensus meeting was very useful in determining the challenges to the proposed RCT. It provided a forum to address and deliberate on several challenging topics related to a future study design. Moreover, the meeting enabled us to gauge support and engagement for the proposed trial, extending beyond the insights from focus groups and interviews. While the relatively small number of stakeholders available for voting is a limitation, the view and results of the stakeholder group were consistent with those echoed in the WP2 focus group interviews and WP3 clinician interviews, reinforcing the validity of the consensus reached.

## Supporting information


**Table S1** Poll questions designed to assess the acceptability and feasibility of a randomized controlled trial of intervention *vs* expectant in the management of selective fetal growth restriction (sFGR) in monochorionic twin pregnancies
**Table S2** Stakeholders who participated in the consensus meeting
