# Supplementary material for: Feasibility and acceptability of randomized controlled trial of intervention vs expectant management for early‐onset selective fetal growth restriction in monochorionic twin pregnancy
Source: Ultrasound Obstet Gynecol. 2025 Jan 24;65(6):671–5. doi: 10.1002/uog.29175 (PMC12127716; doi:10.1002/uog.29175)
Supplement: Supplementary file 1 — Table S1 Poll questions designed to assess the acceptability and feasibility of a randomized controlled trial of intervention vs expectant in the management of selective fetal growth restriction (sFGR) in monochorionic twin pregnancies Table S2 Stakeholders who participated in the consensus meeting [file UOG-65-671-s001.docx]

**Table S1** Poll questions to assess the acceptability and feasibility of a randomized controlled trial (RCT) of intervention *vs* expectant in the management of selective fetal growth restriction (sFGR) in monochorionic twin pregnancies. **#**Consensus threshold was set at a total of 50% of the participants voting ‘yes’.

| **Question** | **Acceptability** | | | **Feasibility** | | | **Consensus met^#^** |
| --- | --- | --- | --- | --- | --- | --- | --- |
|  | **No** | **Unsure** | **Yes** | **No** | **Unsure** | **Yes** | **Yes/No** |
| 1) For cases with Abnormal Umbilical Artery Doppler of the smaller twin - Is it acceptable/feasible to have an RCT without the option of selective termination | 92.9% (13/14) | 7.1% (1/14) | 0% (0/14) | 93.9% (13/14) | 7.1% (1/14) | 0% (0/14) | No, the trial is not acceptable or feasible |
| 2) For cases with Abnormal Umbilical Artery Doppler of the smaller twin with abnormal ductus venosus doppler - Is it acceptable/feasible to have an RCT without the option of selective termination | 71.4% (10/14) | 21.4% (3/14) | 7.1% (1/14) | 85.7% (12/14) | 14.3% (2/14) | 0% (0/14) | No, the trial is not acceptable or feasible |
| 3) For cases with Abnormal Umbilical Artery Doppler of the smaller twin with normal ductus venosus doppler - Is it acceptable/feasible to have an RCT without the option of selective termination | 73.3%  (11/15) | 6.7%  (1/15) | 20.0% (3/15) | 64.3%  (9/14) | 21.4% (3/14) | 14.3% (2/14) | No, the trial is not acceptable or feasible |
| 4) For cases with Normal Umbilical Artery Doppler of the smaller twin - Is it acceptable/feasible to have an RCT without the option of selective termination | 85.7% (12/14) | 7.1% (1/14) | 7.1% (1/14) | 80.0% (12/15) | 13.3% (2/15) | 6.7% (1/15) | No, the trial is not acceptable or feasible |
| 5) For Type I - Is it acceptable/feasible to have an RCT without the option of selective termination? | 88.2% (15/17) | 5.9% (1/17) | 5.9% (1/17) | 88.2% (15/17) | 5.9% (1/17) | 5.9% (1/17) | No, the trial is not acceptable or feasible |
| 6) For Type II - Is it acceptable/feasible to have an RCT without the option of selective termination? | 41.2% (7/17) | 41.2% (7/17) | 17.6% (3/17) | 58.8% (10/17) | 29.4% (5/17) | 11.8% (2/17) | No, the trial is not acceptable or feasible |
| 7) For Type III - Is it acceptable/feasible to have an RCT without the option of selective termination? | 53.3% (8/15) | 26.7% (4/15) | 20.0% (3/15) | 73.3% (11/15) | 13.3% (2/15) | 13.3% (2/15) | No, the trial is not acceptable or feasible |
| 8) Do you think it is feasible to conduct a RCT for management of early onset growth restriction in MC twin pregnancies? |  |  |  | 46.7% (7/15) | 20.0% (3/15) | 33.3% (5/15) | No, the trial is not feasible |

**Table S2** Stakeholders who participated in the consensus meeting

| **Role** | **Region of practice, Country** | **Role in FERN Study** |
| --- | --- | --- |
| Professor of Bioethics | UK | Co-applicant (Ethics) |
| Clinician | Northern Ireland | Co-applicant |
| Sponsorship Officer | UK | Sponsor Representative (University of Liverpool)* |
| Clinician (Chairperson for meeting) | UK | Principal Investigator |
| Clinician (Facilitator for meeting) | Scotland | Chair - FERN Study Steering/Oversight Committee* |
| PPIE representative | UK | Co-applicant (Twins Trust) |
| Clinician | The Netherlands | Collaborator |
| Health Economist | UK | Co-applicant (Health Economics) |
| Clinician | Germany | Collaborator |
| Senior Statistician | UK | Co-applicant (Statistics)* |
| Clinician | UK | Chief Investigator |
| Professor of Biostatistics | UK | Co-applicant (Consensus Development)* |
| PPIE representative | UK | Co-applicant (Twins Trust) |
| Clinician | The Netherlands | Collaborator |
| Clinician | UK | Principal Investigator |
| PPIE representative | UK | Co-applicant (Parent) |
| Research Associate | UK | FERN Core Team - Qualitative |
| Clinician | UK | Principal Investigator |
| Clinician | UK | Principal Investigator |
| Clinician | UK | Collaborator |
| Research Assistant | UK | FERN Core Team – Qualitative* |
| Clinician | UK | FERN Core Team - Study Management |
| Research Coordinator | UK | FERN Core Team - Study Management* |
| Clinician | UK | Principal Investigator |
| Professor of Social Science and Women's Health | UK | Co-applicant |
| Clinician | UK | Co-applicant |
| PPIE representative | UK | Co-applicant (Parent) |
| Professor in Health Research Methodology | UK | Co-applicant (Qualitative Lead) |

*Six people did not participate in voting. PPIE, Patient and Public Involvement and Engagement.
